# Survey of General Practitioner Perspectives on Endometriosis Diagnosis, Referrals, Management and Guidelines in New Zealand

**DOI:** 10.1111/hex.70015

**Published:** 2024-09-02

**Authors:** Katherine Ellis, Alina Meador, Anna Ponnampalam, Rachael Wood

**Affiliations:** ^1^ Department of Chemical and Process Engineering University of Canterbury Christchurch New Zealand; ^2^ Endometriosis New Zealand Christchurch New Zealand; ^3^ School of Population Health, Faculty of Medical and Health Sciences The University of Auckland Auckland New Zealand; ^4^ Department of Physiology, Faculty of Medical and Health Sciences University of Auckland Auckland New Zealand; ^5^ Pūtahi Manawa‐Healthy Hearts for Aotearoa New Zealand Centre of Research Excellence Auckland New Zealand; ^6^ Biomolecular Interaction Centre University of Canterbury Christchurch New Zealand

**Keywords:** clinical care, endometriosis, general practitioners, guidelines, healthcare, routine practice, symptom recognition

## Abstract

**Introduction:**

There is a growing body of literature concerning endometriosis patients' perspectives on the healthcare system and endometriosis care in New Zealand. However, there is little research available on the perspectives of general practitioners (GPs) internationally, and none currently in New Zealand. The purpose of this study is to address New Zealand GPs' understanding of and approach to endometriosis diagnosis, referrals, management and guidelines.

**Methods and Materials:**

An online, anonymous survey was shared with 869 GP clinics and completed by 185 New Zealand‐based GPs regarding their awareness and application of the inaugural 2020 ‘Diagnosis and Management of Endometriosis in New Zealand’ guidelines, their perception of their endometriosis knowledge, the diagnostic value they assign to symptoms, the treatments they recommend and the reasons they refer patients to specialist gynaecologists. Differences between groups were conducted using Chi‐squared tests, and text answers were assessed thematically using inductive, semantic coding.

**Results:**

All 185 GPs had gynaecology consults, and 73% had gynaecology consults every week. Despite 65% being aware of the 2020 guidelines, only 35% overall had read them. Only 52% of GPs considered themselves to know enough about endometriosis for their routine practice. The most common treatment to be considered first line was intrauterine contraceptive devices (IUDs; 96%), whereas the most common alternative treatment recommended was exercise (69%). The most common reason for referral to specialist care was the failure of all attempted treatments (84%).

**Conclusions:**

Many of the study's results align with current New Zealand and international endometriosis guidelines, particularly the prioritisation of progestin‐only therapies, the reduced emphasis on surgical treatment as the first line and the low rates of alternative treatment recommendations. This study also highlights the need to improve awareness of inappropriate GP recommendations, including long‐term treatment with prescription‐only pain relief such as codeine and pregnancy for symptomatic relief.

**Patient or Public Contribution:**

Two of the authors involved in the design and conduct of the study, data interpretation and manuscript preparation have sought care for endometriosis.

**Trial Registration:**

NA

## 1 Introduction

The complexity of endometriosis makes its treatment in primary care challenging. Endometriosis is characterised by the presence of cells similar to the lining of the uterus in locations outside of the uterus [1]. Endometriosis has a wide range of symptoms, including, but not limited to, chronic pelvic pain (CPP), painful periods, ovulatory pain, painful sex, gastrointestinal symptoms and infertility [2, 3, 4]. Endometriosis does not have a unique symptom profile [5], is not always identifiable with imaging modalities (although this is advancing), is not reliably diagnosable using blood tests and has limited predictive value from clinical histories. The gold standard remains surgical removal with histology [6]. For primary care practitioners, the identification of endometriosis symptoms is possible, but these symptoms are also shared by gastrointestinal, musculoskeletal, psychological and other gynaecological conditions [7]. Adding to the complexity for doctors is the fact that there is a growing identification of CPP as a potentially debilitating syndrome in the absence of endometriotic lesions [7].

### 1.1 Current Research in New Zealand

In New Zealand, there is a growing body of literature about the experiences of endometriosis patients navigating primary and secondary care, with reported average delays to diagnosis of up to 10 years [4, 8]. New Zealand patient journeys frequently contain symptom normalisation, trivialisation and dismissal; long diagnostic delays; poor awareness at symptom onset; challenging interactions with medical practitioners; negative influences of the condition on their work, study and family lives; severe pain; and intense emotions at diagnosis, primarily relief [2, 4, 9, 10]. These trends are also reflected internationally in the literature [11, 12, 13]. Conversely, there is limited literature on the perspectives and experiences of general practitioners (GPs) in navigating the diagnosis, management and referral of endometriosis patients in New Zealand.

### 1.2 Influence of Medical Practitioners on Patients

Medical practitioners’ level of knowledge can fundamentally shape patient experiences and diagnostic journeys. In an online survey of participants from 38 countries, when patients were asked about what they would like to share about their experiences with endometriosis, 222 out of 997 highlighted healthcare practitioners as being common barriers or facilitators of care [14]. The most common point raised by this cohort of 222 patients was doubts about the technical competence of their doctors in their endometriosis care, with practitioners frequently described as ‘incompetent’, ‘unskilled’ and ‘unknowledgeable’. Patients felt that there were failings in medical education relating to endometriosis, and conversely, competent practitioners could change their lives through expertise and validation [14].

### 1.3 New Zealand Healthcare Context

Primary care services, such as GP clinics, are the health services New Zealanders interact with most frequently [15]. Similar to other countries with public health systems, such as Australia, GPs are the gatekeepers for accessing public gynaecology services and specialists, as patients require a referral to receive this care publicly [16]. New Zealand also has an agency called Pharmac, which decides which medicines will be funded by the public system and manages the budget for these medicines. Medicines that are not funded by Pharmac can still be available in New Zealand (following approval by the regulatory body Medsafe) [17]. However, they will need to be entirely self‐funded by the individual, whereas medications funded (or partially funded) by Pharmac that are prescribed are available at lower costs for patients under the public health system [18].

### 1.4 International Studies With GPs

In an Iranian study of six gynaecologists and 12 patients, both groups highlighted the difficulty of endometriosis diagnosis given the limitations of diagnostic techniques. Both groups reported frustration at the lack of dependability of physical examinations or blood tests to confirm the presence of endometriosis [19]. Thirteen Norwegian doctors who worked with female infertility patients considered endometriosis one of the three most common and difficult‐to‐diagnose and treat clinical diseases [20]. Similarly, a Western Australian study with nine GPs identified that the challenges in endometriosis care included symptom complexity, clinician experience and awareness, health literacy, limited exposure to endometriosis in education and services access [21]. In an English study with 42 GPs, the process of diagnosing and managing endometriosis was similarly described as complicated by a wide range of factors, including but not limited to diagnostic difficulty, limited access to secondary care and high GP workloads [22]. Therefore, endometriosis‐related qualitative studies about GPs indicate that there are immense difficulties in the provision of diagnosis and treatment for endometriosis patients from the practitioner's perspective. There are also data to indicate GP knowledge regarding endometriosis can be fallible. In a survey of 101 Dutch GPs [23], the GPs answered 16.6 ± 2.4 out of 28 endometriosis knowledge questions correctly. After correction for guessing, the mean score was 46.1% [23], highlighting the knowledge gap that participating GPs had regarding endometriosis. In fact, amongst these GPs, 87.4% felt they needed more education regarding endometriosis [23].

Overall, there is limited availability of research regarding GP perspectives on endometriosis care, and the available studies are predominantly qualitative, which are limited in sample size due to the methodologies used. Therefore, further quantitative research with larger representative samples of practising GPs is needed to complement the existing body of qualitative research.

### 1.5 Endometriosis Guidelines

Clinical guidelines are generally expected to improve care consistency, as regardless of which medical practitioner sees a patient on a certain day and where they are seen, the approach taken should be the same [24]. Guidelines can also reassure medical practitioners about the appropriateness of decisions they make [24]. In New Zealand, inaugural guidelines were released in 2020 titled the ‘Diagnosis and Management of Endometriosis in New Zealand’, which is not classified as a formal clinical guideline. This guideline's intention was to promote awareness of endometriosis symptoms, empower medical practitioners to make suspected diagnoses and improve access and health outcomes for patients [25]. There are also international endometriosis guidelines that are freely available and can be used by medical practitioners around the world. One of the most comprehensive is the 2022 guideline developed by the European Society of Human Reproduction and Embryology (ESHRE) Endometriosis Guideline Development Group, which contains over 100 recommendations for endometriosis best practices [3].

Regularly updating guidelines also makes sure that clinicians have evidence‐based guidelines to support their decision‐making. The provision of outdated care can damage patient trust in the medical practitioners who provide their care [24]. International best practice states that clinical guidelines should be updated every 3–5 years [26]. This timeline indicates that the New Zealand endometriosis guidelines need to be updated to ensure they remain up to date with best practices and can also be adapted to fill knowledge and needs gaps of both patients and GPs. There has not been any public feedback on the New Zealand guidelines to date assessing GP perspectives on their effectiveness or usefulness.

The purpose of this study is to characterise the perspectives, attitudes and approaches of GPs in New Zealand regarding endometriosis care. This study will also assess how this may or may not have been influenced by the inaugural 2020 ‘Diagnosis and Management of Endometriosis in New Zealand’ guidelines and what may merit inclusion in future iterations.

## 2 Materials and Methods

### 2.1 Survey Design

The survey (File S1) was adapted from a 2021 study by Roullier et al. for GPs in France with questions regarding frequency of consultations, gynaecology training, sufficient knowledge of endometriosis, awareness of national endometriosis guidelines, percentage of individuals affected by endometriosis, symptoms suggestive of endometriosis, point of referral to specialist care and first‐line treatments for endometriosis [27]. The purpose of utilising this questionnaire as a base was to allow for comparison between the studies. Additionally, in the current survey, GPs were asked whether they had read the guidelines released in 2020, and found them useful for their practice and which lifestyle features and non‐traditional treatments they recommended to their patients with endometriosis symptoms.

### 2.2 Ethics Approval

The design and approach to the study were reviewed and approved by the University of Canterbury Human Research Ethics Committee (ref: HREC 2023/25). At the start of the survey, the information sheet was displayed with the message that completing the survey would be considered as giving consent to participate and that all survey answers would remain anonymous.

### 2.3 Recruitment

To recruit GPs, an invitation with the survey link and information sheet was emailed to the managers of 869 clinics in New Zealand using publicly available clinic email addresses from clinic websites and HealthPages (an online directory of health services). The clinic managers were asked to forward the email to their clinic's GPs. The survey was estimated to take a maximum of 5 min. There was no reimbursement for participation. It is unknown how many GPs in total received the invitation. According to a 2022 estimate, there are approximately 3850 GPs in New Zealand [28]. Two follow‐up emails were sent to each clinic over the course of 6 months (August 2023 to January 2024) in approximately 2‐month intervals. Two hundred and fifty people opened the link, 225 partially completed the questionnaire and 185 finished the questionnaire and were included in the analysis.

### 2.4 Data Analysis

All statistical analyses were conducted using Qualtrics StatsIQ, with all tests carried out with a confidence level of 95% (*α* = 0.05). The categorical variables were assessed using Pearson's *χ* ^2^ tests (or Fisher's exact test for sample sizes below five). The null hypothesis in all cases was that there were no differences between the groups. One open‐text question was coded in an inductive, iterative thematic manner [29]. Initially, semantic codes were generated according to the explicit meaning of the answers of the 42 participants who provided responses to the open‐ended text question. Subsequently, sentiments were grouped according to common meanings until all remaining themes were distinct.

## 3 Results

### 3.1 Participant Cohort

Of the 185 GPs who completed the survey, 72.4% identified themselves as female (Table 1). Amongst the female cohort, there was a greater proportion of 40–49‐year‐olds than in the male cohort (*p* < 0.05) and a lower proportion over the age of 60 (*p* < 0.001). There were no significant differences in the sex of participants based on region or rurality of practice, location of training or the attainment of further gynaecology training. There were significant sex differences in the frequency of gynaecology consults. Female GPs were significantly less likely than male GPs to have gynaecology consults either less than once per month (*p* < 0.01) or several times a month (*p* < 0.001), and were significantly more likely to have several gynaecology consults per week (*p* < 0.05) or every day (*p* < 0.001).

**Table 1:** Participant characteristics.

Characteristic	*N* (%)
*Gender identity*	*N* = 185
Female	134 (72.4)
Male	48 (25.9)
Prefer not to say (PNS)	3 (1.6)
*Age*	*N* = 185
20–29	4 (2.2)
30–39	44 (23.8)
40–49	47 (25.4)
50–59	47 (25.4)
60+	43 (23.2)
*Region of practice (multiple selections possible)*	*N* = 185
Northland/Te Tai Tokerau	6 (3.2)
Auckland/Tāmaki Makaurau	43 (23.2)
Bay of Plenty/Te Moana‐a‐Toi	11 (5.9)
Waikato	6 (3.2)
Taranaki	1 (0.5)
Gisborne/Te Tairāwhiti	3 (1.6)
Hawke's Bay/Te Matau‐a‐Māui	7 (3.8)
Manawatū‐Whanganui	8 (4.3)
Wellington/Te Whanganui‐a‐Tara	37 (20.0)
Tasman/Te Tai‐o‐Aorere–Nelson/Whakatū	7 (3.8)
Marlborough/Te Tau Ihu‐o‐te‐Waka	1 (0.5)
West Coast/Te Tai Poutini	1 (0.5)
Canterbury/Waitaha	33 (17.8)
Otago/Ōtākou	15 (8.1)
Southland/Murihiku	6 (3.2)
*Location of training (multiple selections possible)*	*N* = 185
University of Auckland/Waipapa Taumata Rau	43 (23.2)
University of Otago/Te Whare Wānanga o Otāgo	83 (44.9)
UK and Europe	48 (25.9)
Australia	9 (4.9)
Asia	8 (4.3)
North America	7 (3.8)
Africa	5 (2.7)
PNS	1 (0.5)
*Rurality of practice*	*N* = 185
Rural	14 (7.6)
Semi‐rural	29 (15.7)
Urban	141 (76.2)
PNS	1 (0.5)
*Further gynaecology training*	*N* = 185
No	50 (27.0)
Gynaecology internship	27 (14.6)
Gynaecology continuing medical education	43 (23.2)
Gynaecology diploma or similar	93 (50.3)
*Time since gynaecology training*	*N* = 135
Less than 3 years	42 (31.1)
Between 3 and 6 years	21 (15.6)
Between 7 and 9 years	17 (12.6)
More than 10 years	55 (40.7)
*Frequency of gynaecology consults*	*N* = 185
Every day	71 (38.4)
Several per week	63 (34.1)
Several per month	45 (24.3)
Less than once per month	6 (3.2)
Never	0 (0.0)

### 3.2 The Diagnosis and Management of Endometriosis in New Zealand Guidelines

The ‘Diagnosis and Management of Endometriosis in New Zealand’ guidelines [30] were released in 2020 at the onset of the COVID‐19 pandemic. The guidelines were released on 2 March 2020 [31], and New Zealand first went into total lockdown 23 days later [32]. Of the 185 GPs, 119 (64.9%) were aware of the guidelines, but only 65 (35.1%) reported that they had read them. The burdens placed on the healthcare system during the pandemic may have left medical practitioners time‐poor, resulting in the low uptake of reading the guidelines. Amongst the 65 GPs who had read the guidelines, 89.2% considered the guidelines useful for their practice. There were no differences in the proportions who had read the guidelines, or who found them useful for their practice based on sex, age, attainment of further gynaecological training or frequency of gynaecology consults.

The New Zealand guidelines contain recommendations for the recognition of endometriosis, preliminary diagnostic investigations and the point to transition patients from primary care into specialist gynaecological management [31]. In an open‐text question about perspectives on the 2020 guidelines, the most frequent sentiment amongst the 42 responses was that there was extremely limited access to public gynaecology services and resources (10 mentions, 23.8% of respondents). Other popular sentiments included that the guidelines had been skimmed (six mentions, 14.2%); that recommended first‐line treatments were unfunded by Pharmac and therefore expensive and inaccessible (as patients would have to pay out of pocket for the full price, rather than paying nothing or having it subsidised) (four mentions, 9.5%); and that it is difficult to have the time to keep up with changes in best practice (9.5%). Furthermore, it was mentioned that it would be better to have clear centralised resources for endometriosis (three mentions, 7.1%); ultrasound is inaccessible for gynaecology (referrals would be denied) (7.1%); private or public access determines patient outcomes (7.1%); and the guidelines represent an inaccessible standard (7.1%):

… the guideline is a work of pure fantasy fiction here.

(Survey Respondent 12)

### 3.3 Knowledge Regarding Endometriosis

When asked if they felt they knew enough about endometriosis for their routine practice, 51.6% of GPs said ‘yes’, 44.6% said ‘somewhat’ and 3.8% said ‘no’ (1 preferred not to say [PNS]). The groups more likely to say ‘yes’ than their comparison groups were those over the age of 60 (*p* < 0.05) and those with daily gynaecological consults (*p* < 0.01). Conversely, the groups more likely to say ‘no’ and consider themselves to not know enough about endometriosis for their routine practice were GPs aged 30–39 (*p* < 0.05), GPs who had not done further gynaecology training (*p* < 0.05), GPs who were not aware of the 2020 guidelines (*p* < 0.01) and GPs who had not read the guidelines (*p* < 0.05).

**Figure 1:**
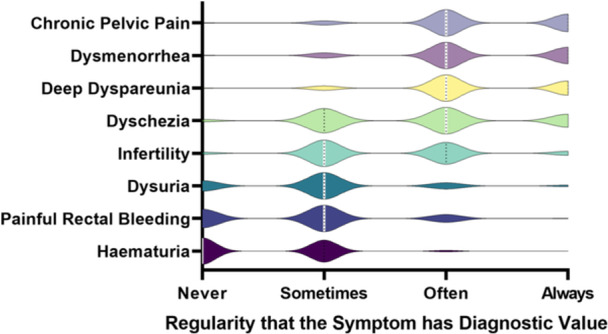
The regularity with which practitioners considered the symptoms of chronic pelvic pain, dysmenorrhoea, deep dyspareunia, dyschezia, infertility, dysuria, painful rectal bleeding and haematuria to have diagnostic value. White lines indicate the median and dotted black lines indicate the interquartile range.

The GPs were asked the diagnostic value they applied to the symptoms of CPP persisting for more than 6 months, dysmenorrhoea (painful periods), deep dyspareunia (painful sex), dyschezia (painful defecation), infertility, dysuria (painful urination), painful rectal bleeding and haematuria (blood in the urine) (Figure 1). Each GP rated the symptoms from 1 to 4 with the options of never, sometimes, often and always having diagnostic value for endometriosis, respectively. The median ratings were that CPP, dysmenorrhoea, deep dyspareunia and dyschezia ‘often’ had diagnostic value (median rating of 3), whereas infertility, dysuria and painful rectal bleeding were considered to ‘sometimes’ have diagnostic value (median rating of 2) and haematuria to ‘never’ have diagnostic value (median rating of 1). There were no significant differences in scores assigned to symptoms based on whether or not the GP had read the 2020 guidelines or had further gynaecology training.

### 3.4 Prevalence of Endometriosis

**Figure 2:**
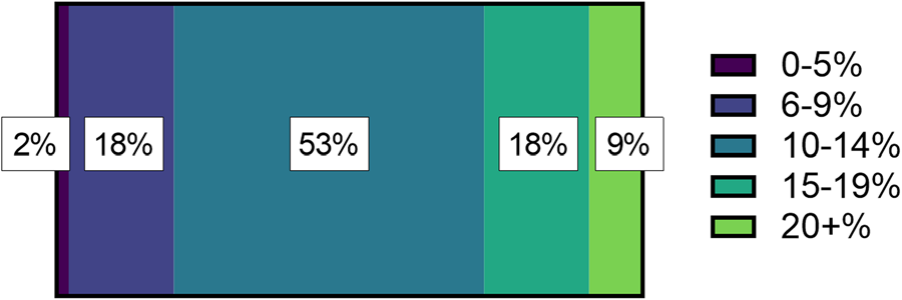
The proportion of GPs that estimated the prevalence of endometriosis amongst women and people presumed female at birth in New Zealand was 0%–5%, 6%–9%, 10%–14%, 15%–19% or over 20%.

The prevalence of endometriosis is frequently accepted as 10% [25, 31] and reports of up to 14% in Australia [33], but the precise prevalence in New Zealand is unknown. When asked what percentage of women and people assigned female at birth they thought had endometriosis in New Zealand, 52.7% of the cohort thought that it was 10%–14% (Figure 2). Those who did further gynaecology training were significantly less likely than those who did not (*p* < 0.05) to think that the prevalence of endometriosis was less than 10%, whereas there were no significant differences between those who had and had not read the 2020 guidelines.

### 3.5 Management of Endometriosis

The treatments considered first line by the majority of GPs (Table 2) were intrauterine contraceptive devices (IUDs; 95.7%), combined oral contraceptive pills (86.5%), progesterone injections (71.9%) and progestin‐only contraceptive pills (69.7%). Progestin‐only contraceptive pills were more likely to be recommended by GPs who had read the New Zealand guidelines (*p* < 0.01), those trained in New Zealand (*p* < 0.001) and those with daily gynaecology consults (*p* < 0.001).

**Table 2:** Perception of first‐line treatments, alternative treatments to recommend and reasons for referral for male and female GPs, GPs who had (FGT+) and had not (FGT−) completed further gynaecology training and GPs who had (RG+) and had not (RG–) read the 2020 Diagnosis and Management of Endometriosis in New Zealand guidelines.^a^

	See footnote^b^	Total (*N* = 185)	Male (*N* = 48)	Female (*N* = 134)	FGT+ (*N* = 135)	FGT− (*N* = 50)	RG+ (*N* = 65)	RG− (*N* = 120)
*What are the first‐line treatments for symptomatic endometriosis?*								
Intrauterine contraceptive devices	+	177 (95.7%)	45 (93.8%)	129 (96.3%)	129 (95.6%)	47 (94.0%)	63 (96.9%)	113 (94.2%)
Combined oral contraceptive pills	+	160 (86.5%)	42 (87.5%)	116 (86.6%)	115 (85.2%)	44 (88.0%)	54 (83.1%)	105 (87.5%)
Progesterone injections	+	133 (71.9%)	36 (75.0%)	97 (72.4%)	102 (75.6%)	30 (60.0%)	47 (72.3%)	85 (70.8%)
Progestin‐only contraceptive pills	+	129 (69.7%)	34 (70.8%)	93 (69.4%)	94 (69.6%)	35 (70.0%)	54 (83.1%)**	75 (62.5%)
Implant with etonogestrel	+	92 (49.7%)	29 (60.4%)	63 (47.0%)	69 (51.1%)	22 (44.0%)	35 (53.8%)	56 (46.7%)
Non‐contraceptive progestins	+	86 (46.5%)	27 (56.3%)	57 (42.5%)	66 (48.9%)*	20 (40.0%)	33 (50.8%)	53 (44.2%)
Long‐term treatment with NSAIDs	+	85 (45.9%)	27 (56.3%)	61 (45.5%)	62 (45.9%)	23 (46.0%)	30 (46.2%)	55 (45.8%)
Surgery	—	74 (40.0%)	22 (45.8%)**	45 (33.6%)	52 (38.5%)	22 (44.0%)	19 (29.2%)*	55 (45.8%)
Gonadotrophin‐releasing hormone treatment	—	45 (24.3%)	14 (29.2%)	30 (22.4%)	38 (28.1%)*	7 (14.0%)	13 (20.0%)	32 (26.7%)
Long‐term treatment with prescription‐only pain relief	—	12 (6.5%)	5 (10.4%)	6 (4.5%)	8 (5.9%)	4 (8.0%)	3 (4.6%)	9 (7.5%)
*Which of the following do you recommend to patients who present with endometriosis symptoms?*								
Exercise	+	128 (69.2%)	28 (58.3%)	98 (73.1%)	96 (71.1%)	32 (64.0%)	53 (81.5%)**	75 (62.5%)
Counselling/talk‐based therapy		72 (38.9%)	12 (25.0%)*	59 (44.0%)	56 (41.5%)	16 (32.0%)	28 (43.1%)	44 (36.7%)
Chronic pain clinic	+	70 (37.8%)	13 (27.1%)	56 (41.8%)	51 (37.8%)	19 (38.0%)	27 (41.5%)	43 (35.8%)
Physiotherapy	+	67 (36.2%)	14 (29.2%)	52 (38.8%)	48 (35.6%)	19 (38.0%)	27 (41.5%)	40 (33.3%)
Diet changes	+	48 (25.9%)	7 (14.6%)*	39 (29.1%)	36 (26.7%)	12 (24.0%)	24 (36.9%)*	24 (20.0%)
Meditation		45 (24.3%)	16 (33.3%)	28 (20.9%)	32 (23.7%)	13 (26.0%)	18 (27.7%)	27 (22.5%)
Weight loss		33 (17.8%)	11 (22.9%)	21 (15.7%)	23 (17.0%)	10 (20.0%)	13 (20.0%)	20 (16.7%)
Transcutaneous electrical nerve stimulation machine	+	33 (14.6%)	3 (6.3%)	23 (17.2%)	19 (14.1%)	8 (16.0%)	13 (20.0%)	14 (11.7%)
Acupuncture		25 (13.5%)	8 (16.7%)	17 (12.7%)	20 (14.8%)	5 (10.0%)	8 (12.3%)	17 (14.2%)
Pregnancy		12 (6.5%)	6 (12.5%)	6 (4.5%)	10 (7.4%)	2 (4.0%)	4 (6.2%)	8 (6.7%)
Medicinal cannabis		11 (5.9%)	5 (10.4%)	6 (4.5%)	9 (6.7%)	2 (4.0%)	3 (4.6%)	8 (6.7%)
Supplements		9 (4.9%)	2 (4.2%)	6 (4.5%)	7 (5.2%)	2 (4.0%)	2 (3.1%)	7 (5.8%)
Botox		3 (1.7%)	1 (2.1%)	1 (0.7%)	1 (0.7%)	2 (4.0%)	1 (1.5%)	2 (1.7%)
*When do you refer a patient to a specialist gynaecologist?*								
If all attempted first‐line treatments for endometriosis symptoms have failed	+	155 (83.8%)	36 (75.0%)*	117 (87.3%)	114 (84.4%)	41 (82.0%)	55 (84.6%)	100 (83.3%)
For treatment of a fertility issue	+	152 (82.2%)	38 (79.2%)	112 (83.6%)	117 (86.7%)**	35 (70.0%)	57 (87.7%)	95 (79.2%)
If initial treatment for endometriosis symptoms fails	+	127 (68.6%)	37 (77.1%)	87 (64.9%)	89 (65.9%)	38 (76.0%)	36 (55.4%)**	91 (75.8%)
If the results of a clinical examination are abnormal		94 (50.8%)	22 (45.8%)	71 (53.0%)	70 (51.9%)	24 (48.0%)	34 (52.3%)	60 (50.0%)
If the patient is having to miss study and/or work because of symptoms		85 (45.9%)	28 (58.3%)*	54 (40.3%)	57 (42.2%)	28 (56.0%)	27 (41.5%)	58 (48.3%)
Patient has a confirmed diagnosis of endometriosis and presents with symptoms		69 (37.3%)	22 (45.8%)	44 (32.8%)	51 (37.8%)	18 (36.0%)	25 (38.5%)	44 (36.7%)
Immediately upon request by the patient		48 (25.9%)	15 (31.3%)	30 (22.4%)	34 (25.2%)	14 (28.0%)	16 (24.6%)	32 (26.7%)
As soon as you suspect endometriosis is likely		22 (11.9%)	10 (20.8%)*	12 (9.0%)	16 (11.9%)	6 (12.0%)	8 (12.3%)	14 (11.7%)
When treatment has to be initiated for endometriosis symptoms		12 (6.5%)	4 (8.3%)	8 (6.0%)	10 (7.4%)	2 (4.0%)	2 (3.1%)	10 (8.3%)
Following blood tests		6 (3.2%)	3 (6.3%)	3 (2.2%)	5 (3.7%)	1 (2.0%)	2 (3.1%)	4 (3.3%)

^a^ Comparisons were done between groups (male and female, FGT+ and FGT– and RG+ and RG–), and significance is shown by * for *p* < 0.05 and ** for *p* < 0.01 in the left column of the comparison group.

^b^ Suggestions by GPs accompanied by a ‘+’ indicate that these are indicated in the 2020 Diagnosis and Management of Endometriosis in New Zealand guidelines as a first‐line approach, potential non‐pharmacological strategy or reason for referral for specialist attention. Suggestions with a ‘–’ indicate that this strategy is dissuaded as the first line in primary care in the 2020 guidelines. Suggestions without a ‘+’ or ‘–’ are not explicitly referred to within the guidelines as a non‐pharmacological strategy or reason for referral.

The only alternative treatment method for endometriosis that was recommended by a majority of GPs was exercise (69.2%; Table 2). GPs with daily gynaecology consults were more likely to recommend diet changes (*p* < 0.05) and physiotherapy (*p* < 0.05) than GPs with less frequent consults. GPs who were aged 40–49 were less likely to recommend pregnancy (*p* < 0.05), whereas those aged 30–39 were more likely to recommend weight loss (*p* < 0.05), physiotherapy (*p* < 0.05) and chronic pain clinics (*p* < 0.05). GPs over 60 were more likely to recommend pregnancy (*p* < 0.05) and less likely to recommend physiotherapy (*p* < 0.01), meditation (*p* < 0.05) and chronic pain clinics (*p* < 0.05). Those trained in New Zealand were more likely to recommend physiotherapy than those trained overseas (*p* < 0.05).

The most commonly reported reasons GPs would refer patients to specialist gynaecology services (Table 2) were the failure of all first‐line treatments (83.8%), treating fertility issues (82.2%), failure of initial treatment (68.6%) and abnormal clinical examinations (50.8%). GPs with daily gynaecology consults were less likely to refer patients who missed study and/or work because of symptoms (*p* < 0.05). Those with several gynaecology consults per month were more likely to refer after blood tests (*p* < 0.05). GPs with less than one gynaecology consult per month were less likely to refer as a result of fertility issues (*p* < 0.05).

## 4 Discussion

### 4.1 Limitations

This survey was opt‐in and anonymous. This means that GPs, who are very busy individuals, had to receive the email invitation to participate from their clinic manager and then decide to take part. Although 869 clinics were contacted to invite their GPs to participate, only 185 GPs chose to complete the survey in full. The GPs who chose to take the time to participate and complete the survey will likely differ in their characteristics from those who chose not to. The results in this study may be biased towards the perspectives and attitudes of GPs who may have a greater interest in endometriosis and therefore may be more interested in participating in a project about endometriosis. This may have biased results towards GPs with a better perception of their endometriosis knowledge, who may be more likely to have read the guidelines and taken part in further gynaecology training. This bias may therefore make this GP sample more aware of up‐to‐date recommendations about endometriosis compared to GPs generally. This sample represents approximately 4.8% of the GPs in New Zealand [28], and a larger sample would likely have been more representative of the overall GPs in New Zealand.

Participants in this study were more likely than the general population of GPs to be female, as in a 2022 survey by the Royal New Zealand College of General Practitioners that was completed by 70% of the College's members (*N* = 3356); only 58% of respondents were female [34], versus 72.4% in the present study. In the Royal College's survey, 62% of GPs got their medical degree in New Zealand, which is similar to this survey's cohort, where 63.6% of GPs studied at one of New Zealand's two medical schools. The age proportions in this cohort are similar to the College's survey proportions, which had quartile points at approximately 39, 52 and 61 years [34].

### 4.2 GP Knowledge

In this cohort, only 52% of GPs felt certain that their knowledge regarding endometriosis was sufficient for their routine practice. The 2021 survey by Roullier et al., which involved 102 GPs in France, used similar questions to those included in this study [27]. Differences between the GPs in the French cohort and this New Zealand cohort are that the New Zealand cohort had a higher proportion of female GPs (72.4% vs. 54.9%), GPs over 50 (48.6% vs. 30.4%), GPs with gynaecology diplomas (50.3% vs. 11.7%) and fewer GPs practising in rural areas (7.6% vs. 31.4%). In the Roullier et al. study, only one in four GPs felt they knew enough about endometriosis for their routine practice (options to select were binary, there was no option to select ‘somewhat’ as there was in this study) [27]. In the Roullier cohort, 71.6% of GPs had less than one gynaecological consult per week [27], compared to 27.5% in this cohort, which may account for the differences in the proportion reporting insufficient knowledge. In both studies, the proportion that felt they had sufficient knowledge regarding endometriosis was significantly increased amongst those with further gynaecology training [27].

In this study, the symptoms of CPP, dysmenorrhoea, deep dyspareunia and dyschezia were given median ratings of 3/4 (‘often’) for diagnostic value; infertility, dysuria and painful rectal bleeding were rated 2/4 (‘sometimes’); and haematuria was rated 1/4 (‘never’) for having diagnostic value. The Roullier et al. study results were similar, with CPP, intense dysmenorrhoea and severe dyspareunia receiving median ratings of ‘often’ prompting consideration of endometriosis, whereas catamenial painful defecation and urinary function disorders were rated as ‘sometimes’ prompting consideration. The key difference between the studies is the French cohort considered fertility disorders to ‘often’ have diagnostic value, whereas the New Zealand GPs considered infertility to ‘sometimes’ have diagnostic value.

All symptoms that were rated in this study, bar CPP, are listed in the ESHRE guidelines as individually warranting suspicion of endometriosis even in the absence of the other symptoms, particularly when cyclical or catamenial in nature [3]. This would indicate these symptoms should often or always be considered to have diagnostic value. Meanwhile, in the New Zealand guidelines, the symptoms that alone or as a combination merit consideration of endometriosis from menarche onwards include dysmenorrhoea limiting quality of life, dyspareunia, unexplained sub‐fertility and unexplained urinary and gastrointestinal symptoms [30]. In the New Zealand guidelines, early recognition of suspicious symptoms is considered a key aim [30], but there was no difference in the diagnostic value assigned to symptoms between those who had and had not read the guidelines. This finding indicates an area for consideration in strengthening the awareness of the range of endometriosis symptoms within the New Zealand guidelines so that when they present in practice, endometriosis is considered as part of that differential assessment.

Endometriosis prevalence estimates can be variable and are often reported as 10% [25, 31] but can range anywhere between 0.11% [35] and 16.8% [36] in general presumed‐female‐at‐birth population cohorts depending on the methodology. There is no estimate reported within the New Zealand endometriosis guidelines [30]. Amongst the GPs in this study, 19.7% thought that the prevalence was less than 10%, 52.7% thought it was in the ‘correct’ range of 10%–14% and 27.4% thought the prevalence exceeded 15%. In a survey of 101 Dutch GPs, they encountered an average of 2.8 ± 2.5 individuals annually they suspected of having endometriosis, which is lower than you would anticipate if 10% of the presumed‐female‐at‐birth population had the condition [25]. New Zealand‐based research on endometriosis prevalence should be conducted so GPs can be equipped with a reasonable expectation of the proportion of their patients they can expect to have endometriosis.

### 4.3 First‐Line Treatments

Prior research with practitioners has highlighted difficulties in endometriosis diagnosis and management [19, 20, 21]. These difficulties are reflected in the repeated reports that amongst patients with confirmed endometriosis in New Zealand, there are delays to diagnosis of at least 8 years from symptom onset [2, 4, 8]. These delays make the decisions around which first‐line treatments to offer to patients without specialist input particularly important as there will likely be long periods where these treatments are the only support possible for symptom management.

In this study, the results showed a focus on progestin‐only therapies and the dissuasion of laparoscopy as a first line treatment for endometriosis symptoms. Both fundings align with recommendations from the Diagnosis and Management of Endometriosis in New Zealand guidelines [30]. The ESHRE guidelines also strongly recommended first‐line progestin therapies and dissuade diagnostic laparoscopies [3]. The dissuasion of surgical interventions as first line is a recent shift, as in a review of eight guidelines from around the world published between 2010 and 2018, all listed excision for endometriomas, and seven listed excision in general, as first‐line treatment of endometriosis pain [37].

Concerningly, 6.5% of the 185 GPs reported that they considered long‐term treatment with prescription‐only pain relief (e.g., codeine) to be the first line. Opioid pain medications are not appropriate for endometriosis treatment [38], and awareness of negative impacts (including side effects and addiction risks) is becoming increasingly publicly recognised [39] and is actively dissuaded in the New Zealand 2020 guidelines [30]. Therefore, further awareness is needed to prevent the use of opioids as first‐line treatment.

All first‐line treatments assessed in both the Roullier et al. study and this cohort were rated as first line by much higher proportions of this survey's participating GPs. The largest difference was IUDs, which were considered first line by 95.7% of this survey's GPs, but only 29.4% of the 102 GPs in the Roullier et al. study. Meanwhile, there was only a 14.8% difference in the proportion of GPs considering progestin‐only contraceptive pills first line in the two studies [27]. The high number of GPs in this study considering hormonal therapies first line may conflict with patient preferences, as prior New Zealand research found patient perspectives on the effectiveness of IUDs for endometriosis to be conflicting. In a study of 50 endometriosis patients’ experiences in New Zealand, IUDs were the treatment the second highest number of patients wished they had never tried, and 50.0% of users found them effective for symptomatic relief [2].

### 4.4 Alternative Therapeutics

Non‐pharmacological strategies for endometriosis recommended by the New Zealand guidelines are diet (recommended by 25.9% in this study), exercise (69.2%), sleep, transcutaneous electrical nerve stimulation machines (14.6%), pain psychology and specialist physiotherapy (36.2%) [30]. The five least recommended alternative treatments in this study were acupuncture (13.5%), which has some evidence for usefulness for other chronic pain conditions but limited evidence for endometriosis [3, 40]; pregnancy (6.5%) which is dissuaded in the ESHRE guidelines [3] due to poor evidence of reducing the extent of the disease [9, 41]; medicinal cannabis (5.9%) which has had positive reports of pain reduction in New Zealand studies [2, 42]; supplements (4.9%) for which data supports potential improvements but overall differences can be weak relative to placebos [43]; and botulinum toxin (botox, 1.6%) that has had some emerging research to support its use in endometriosis [44, 45].

Acupuncture, physiotherapy, transcutaneous electrical nerve stimulation machines and electrotherapy, psychological interventions, specific diets or Chinese medicine are considered in the 2022 ESHRE guidelines to have insufficient evidence to be recommended for pain relief or improved quality of life [3]. These guidelines do cautiously recommend exercise, as exercise even if not specifically therapeutic, is considered as part of a healthy lifestyle, but further endometriosis‐specific research is still needed [3]. As further evidence is produced for or against these alternative therapies, it will be important to monitor the uptake of alternative therapies by GPs and patients alike and whether these recommendations will continue to align with the recommendations of the New Zealand guidelines and ESHRE.

### 4.5 Reasons for Specialist Referral

In the New Zealand endometriosis guidelines, referral to secondary gynaecological specialist care is recommended if symptoms have not been able to be controlled within primary healthcare management [30], such as with hormonal treatments. This is consistent with the top reason for referral in this cohort being the failure of all attempted treatments to control symptoms (83.8%). There were different patterns for referral to specialist gynaecological care between the GPs in this study and the GPs in the Roullier et al. French cohort. In this cohort, referrals were likely to be made for the treatment of fertility (82.2%) or initial treatment failing (68.6%), compared to 52.9% and 21.6%, respectively, in the French cohort [27]. Conversely, only 11.9% of this cohort would refer to a gynaecologist as soon as endometriosis was suspected, and 6.5% would refer at treatment initiation, whereas 52.0% and 26.5%, respectively, of GPs in the French cohort would do the same [27]. These differences may relate to differences in the referral processes and healthcare systems between the two countries. In France, there are approximately 23.0 obstetrician gynaecologists (OBGYNs) per 100,000 females [46, 47], whereas in New Zealand, there are only approximately 13.6 per 100,000 females [28, 48], which has been consistent for over a decade [49]. The low per capita prevalence of OBGYNs may explain the perception in this cohort that gynaecology services are extremely limited. The discrepancy in OBGYNs between France and New Zealand may mean that in New Zealand, GPs may be more likely to expect that for a referral to be accepted, the patient needs to be further in their endometriosis journey, such as having the initial treatment failed, rather than only initiating treatment. Conversely, the French GPs may have been more interested in earlier referral points (e.g., upon treatment initiation) due to greater availability of OBGYNs, rather than waiting until later in that journey.

### 4.6 The Diagnosis and Management of Endometriosis in New Zealand Guidelines

The 2020 Diagnosis and Management of Endometriosis in New Zealand guidelines released by the Ministry of Health were considered useful for their practice by 89.2% of the GPs who had read them. This study indicates that GPs find these guidelines to be useful and that they represent a standard of care difficult to attain due to limited access to gynaecology services and resources, including ultrasound and treatment services. Amendment of these guidelines should be undertaken in tandem with health services improvements that address gynaecology access issues, in collaboration with both patients and medical practitioners. Emerging conceptualisations for improved clinical guideline development and uptake indicate development should be iterative and with the buy‐in and input of a full range of stakeholders [50, 51]. As there has been sufficient time since the first iteration of the endometriosis guidelines to justify their update and redevelopment, it would be apt to incorporate the perspectives and feedback of GPs into the next guidelines.

Fundamental to the success of the next iteration of the guidelines will be their roll‐out. The present iteration had only been read by 35.1% of the GPs in this cohort. The low readership limits the impact of guidelines on primary care in New Zealand. To ensure that the changes and improvements in the next guidelines translate into care, it must be ensured that the guidelines are palatable for GPs to read and well‐advertised to ensure GPs know that they are available and should be read.

## 5 Conclusions

In this study, 185 GPs from around New Zealand took part in a survey about the New Zealand guidelines and the current approach they take to caring for endometriosis patients. Only 35% of GPs had read the New Zealand Endometriosis guidelines, and a slim majority (52%) felt sure they knew enough about endometriosis for their routine practice. Overall, the approach taken by GPs (prioritising progestin therapies and dissuading surgery as the first line) aligns with both the New Zealand and international ESHRE guidelines for best practice. However, the presence of GPs in the study who recommend prescription‐only pain relief as first line and recommend pregnancy to patients with endometriosis symptoms is concerning. These trends indicate that there is a place for updated guidelines in New Zealand. Any updated guidelines should be affiliated with a well‐designed distribution plan to significantly increase and incentivise readership amongst GPs. All participating GPs conducted gynaecology consults in their practice and should therefore be appropriately versed in endometriosis, which with an estimated prevalence of 10% amongst presumed‐female‐at‐birth individuals, is a common condition.

## Author Contributions

**Katherine Ellis:** conceptualisation, investigation, writing–original draft, visualisation, formal analysis. **Alina Meador:** conceptualisation, investigation, writing–review and editing. **Anna Ponnampalam:** supervision, writing–review and editing, conceptualisation. **Rachael Wood:** supervision, writing–review and editing, conceptualisation.

## Ethics Statement

The design and approach to the survey study were reviewed and approved by the University of Canterbury Human Research Ethics Committee (ref: HREC 2023/25).

## Consent

At the start of the survey, the information sheet was displayed with the message that completing the survey would be considered consent to participate and that all survey answers would remain anonymous.

## Conflicts of Interest

Katherine Ellis is the Research Coordinator for Endometriosis New Zealand. The other authors declare no conflicts of interest.

## Supporting information

Supporting information.

## Data Availability

Anonymised data sets will be made readily available by the authors upon reasonable request.
