# Supplementary material for: Survey of General Practitioner Perspectives on Endometriosis Diagnosis, Referrals, Management and Guidelines in New Zealand
Source: Health Expect. 2024 Sep 2;27(5):e70015. doi: 10.1111/hex.70015 (PMC11369013; doi:10.1111/hex.70015)
Supplement: Supplementary file 1 — Supporting information. [file HEX-27-e70015-s001.docx]

Survey Questions:

1. Demographics
   1. Do you identify as:

- Male
- Female
- Gender Diverse
  1. Are you:
- 20-29
- 30-39
- 40-49
- 50-59
- 60+
  1. Which region of New Zealand do you practice in:
- Northland
- Auckland
- Bay of Plenty
- Waikato
- Taranaki
- Gisborne
- Hawke’s Bay
- Whanganui-Manawatu
- Wellington
- Nelson-Tasman
- Marlborough
- West Coast
- Canterbury
- Otago
- Southland
  1. Where have you trained (Multi-choice):
- University of Otago
- University of Auckland
- Australia
- North America
- South America
- Asia
- Africa
- UK & Europe
  1. Is where you practice:
- Rural
- Semi-rural (Definition: “somewhat rural”)
- Urban
  1. After you finished your medical degree, did you do further gynaecological training:
- No
- Yes – Gynaecology internship
- Yes – Gynaecology CME (Continuing Medical Education)
- Yes – Gynaecology Diploma or similar
  1. How frequently do you have gynaecology consults:
- Never
- Less than once per month
- Several per month
- Several per week
- Every day

1. Endometriosis in your Practice
   1. Are you aware of the “Diagnosis and Management of Endometriosis in New Zealand” guidelines released by the Ministry of Health in 2020?

- Yes
- No
  1. Have you read the “Diagnosis and Management of Endometriosis in New Zealand” guidelines released by the Ministry of Health in 2020?
- Yes
- No
  1. (Only for 2.2. = Yes) Do you think the “Diagnosis and Management of Endometriosis in New Zealand” guidelines released by the Ministry of Health in 2020 are useful for your practice?
- Yes
- No
  1. Do you feel you know enough about endometriosis for your routine practice?
- Yes
- Somewhat
- No
  1. What five adjectives would you use to describe patients presenting with endometriosis symptoms?
  2. Please rate the following symptoms out of 4 for diagnostic value for endometriosis.

1 = never has diagnostic value for endometriosis.

2 = sometimes has diagnostic value for endometriosis.

3 = often has diagnostic value for endometriosis.

4 = always has diagnostic value for endometriosis.

- - 1. Dysmenorrhea (painful periods)
    2. Deep dyspareunia (painful sex)
    3. Infertility
    4. Dysuria (painful urination)
    5. Dyschezia (painful defecation)
    6. Chronic pelvic pain (for a period of six months or more)
    7. Painful rectal bleeding
    8. Haematuria (blood in the urine)
  1. What percentage of women (and people assigned female at birth) do you think have endometriosis in New Zealand?
- 0-5%
- 6-9%
- 10-14%
- 15-19%
- 20%+
  1. What are the front-line treatments for symptomatic endometriosis? (Multi-choice)
- Implant with etonogestrel (e.g. Levonorgestrel [Jadelle®])
- Long-term treatment with NSAIDs (e.g. Ibuprofen)
- GnRH (Gonadotrophin-releasing hormone) treatment (e.g. Goserelin [Zoladex®])
- Progestin-only oral contraceptive pills (e.g. Desogestrel [Cerazette®])
- Combined oral contraceptive pills (e.g. norethisterone/ethinylestradiol [Brevinor®])
- Non-contraceptive progestins (e.g. medroxyprogesterone acetate [Provera®])
- Long-term treatment with prescription-only pain relief (e.g. Codeine)
- Surgery
- IUCDS (Intra-uterine contraceptive device (e.g. Levonorgestrel [Mirena® or Jaydess®])
- Progesterone injections (e.g. Medroxyprogesterone acetate [Depo-Provera®])
- Other (Please specify)
  1. Which of the following do you recommend to your patients that present with endometriosis symptoms? (Multi-choice)
- Chronic pain clinic
- Exercise
- Acupuncture
- Counselling/Talk-based therapy
- Pregnancy
- Diet changes
- TENS (transcutaneous electrical nerve stimulation) machines
- Supplements
- Weight loss
- Botox
- Physiotherapy
- Medicinal cannabis
- Meditation
- Other (Please specify)
  1. When do you refer a patient to a specialist gynaecologist? (Multi-choice)
- If the results of a clinical examination are abnormal
- Following blood tests
- For treatment of a fertility issue
- If initial treatment for endometriosis symptoms fails
- If the patient is having to miss study and/or work because of symptoms
- If all attempted first-line treatments for endometriosis symptoms have failed
- When treatment has to be initiated for endometriosis symptoms
- As soon as you suspect endometriosis is likely
- Immediately upon request by the patient
- When the patient has a confirmed surgical or radiological diagnosis of endometriosis and presents with endometriosis symptoms
- Other (Please specify)

1. Would you like to be contacted for a 10-20 minute discussion about endometriosis in your practice?

- Yes – please follow the link to an interest form (which will not be connected with your answers to this survey) and we will send you the information sheet and consent form for this follow-up study
- No
